# Automated RECIST tumor response classification through prompt-guided large language models

**DOI:** 10.1038/s41598-026-54979-y

**Published:** 2026-05-27

**Authors:** Markus Mergen, Felix Busch, Andreas P. Sauter, Daniela Pfeiffer, Marcus R. Makowski, Daniel Spitzl, Florian T. Gassert

**Affiliations:** 1 Department of Diagnostic and Interventional Radiology, Technical University of Munich, School of Medicine and Health, Klinikum rechts der Isar, TUM University Hospital, 81675 Munich, Germany; 2 Munich Institute for Advanced Study, Technical University of Munich, 85748 Garching, Germany; 3 Medical Clinic and Polyclinic II, TUM School of Medicine and Health, TUM University Hospital, Technical University Munich (TUM), Ismaningerstr. 22, 81675 Munich, Germany

**Keywords:** Cancer, Mathematics and computing, Medical research, Oncology

## Abstract

This study investigates whether an entirely offline, general-purpose large language model (LLM) can reliably automate the classification of routine radiology reports according to RECIST (Response Evaluation Criteria in Solid Tumors) guidelines, focusing on how different prompting strategies support accurate, privacy-preserving tumor response assessment without additional model fine-tuning. An offline, in-house implementation of LLaMA-3.3 (70B) was used to classify real-world CT imaging reports from oncology patients. Reports were authored following RECIST-structured reporting but had outcome labels programmatically withheld prior to processing. Three prompting strategies—zero-shot, few-shot, and chain-of-thought prompting—were tested to guide the model in assigning RECIST categories: Baseline (BL), Complete Response (CR), Partial Response (PR), Stable Disease (SD), and Progressive Disease (PD). Model outputs were benchmarked against original expert labels using accuracy, precision, recall, and F1 scores. Across all tested prompting strategies, the LLaMA-3.3 model achieved strong classification performance. The best results were obtained with chain-of-thought prompting, reaching micro F1 scores of 0.81 across all RECIST categories. Overall model predictions aligned well with human expert assessments. Operating entirely offline within hospital infrastructure, the system preserved full compliance with stringent data privacy requirements. Prompt-driven large language models can accurately classify tumor response categories from real-world radiology reports in a scalable, reproducible, and privacy-preserving manner. Offline LLM deployment, combined with optimized prompting strategies, offers a promising approach for automating structured oncology report interpretation, potentially enhancing consistency and efficiency in clinical decision support workflows.

**Supplementary Information:**

The online version contains supplementary material available at 10.1038/s41598-026-54979-y.

## Introduction

Standardized assessment of tumor response is a cornerstone of oncology care, guiding decisions on treatment continuation, clinical trial eligibility, and prognostic evaluation^1^. The Response Evaluation Criteria in Solid Tumors (RECIST) provide a widely accepted framework for categorizing disease progression or response based on imaging studies^1,2^. However, determining RECIST classifications from narrative radiology reports poses a operational challenge; although reports in our dataset followed structured RECIST reporting conventions, the outcome labels were embedded within free-text sections. This required careful parsing and interpretation, which demands clinical expertise and remains susceptible to calculation errors, oversight of specific criteria (e.g., Nadir referencing), and discordance between the measured values and the written conclusion.

Recent advances in artificial intelligence (AI), particularly in natural language processing (NLP), have opened new avenues for automating clinical documentation analysis^3–7^. Large language models (LLMs) such as Llama 3 demonstrate remarkable proficiency in understanding and reasoning over complex medical language, making them well-suited for interpreting nuanced radiology reports^8^. These models are capable of grasping subtle contextual cues, handling variable terminology, and adapting to the heterogeneity found in real-world clinical narratives—features essential for accurately inferring structured information like RECIST classifications.

While prior studies have applied LLMs to extract individual clinical concepts such as diagnoses or measurements from medical text^9–11^, relatively few have examined their potential for more integrative, decision-oriented tasks in oncology^4,12,13^. Furthermore, much of the existing literature has focused on fine-tuned models or synthetic data, limiting generalizability to routine clinical settings^14–16^. Whether today’s general-purpose foundation models can reliably interpret authentic imaging reports and produce valid RECIST assessments remains an open and practically relevant question.

In this study, we evaluate an offline deployment of the Llama-3.3 (70B) model for the classification of real radiology reports according to RECIST criteria. Using a clinical routine dataset with RECIST annotations withheld, we investigate the effectiveness of three prompting strategies—zero-shot prompting, few-shot prompting and chain-of-thought (CoT) prompting—in guiding the model’s decision-making. By benchmarking LLaMA 3.3’s performance against expert-labeled outcomes, we aim to assess the feasibility of prompt-driven NLP in supporting oncology workflows and explore its potential as a privacy-preserving tool for automated, structured report interpretation.

## Materials and methods

This study was approved by the Institutional Review Board of the Technical University of Munich (2024-590-S-CB) and conducted in accordance with all applicable ethical and data protection regulations. The requirement for individual informed consent was waived by the ethics committee due to the retrospective design and the use of fully anonymized clinical data.

A total of 142 radiology reports were included, each corresponding to a clinically documented follow-up examination in patients with solid tumors. Reports were authored by board-certified radiologists and followed internal institutional reporting conventions designed to facilitate the application of RECIST 1.1 criteria. While the official RECIST 1.1 guidelines are primarily defined for response assessment in clinical trials and do not mandate a specific reporting template, our institution utilizes semi-structured templates (comprising dedicated fields for target lesion diameters and separate free-text sections for the final assessment) to standardize the documentation of these measurements in daily oncological practice. These templates include predefined sections for target and non-target lesions, while the final response assessments remain documented in free-text form. Crucially, each structured report utilized in this study contains the relevant longitudinal reference data required by RECIST 1.1. This includes the documentation of baseline measurements for calculating Partial Response (PR) and the smallest recorded measurements (nadir) for determining Progressive Disease (PD). By including these reference values within the current report’s text, the model was provided with the necessary comparative context to infer the correct category without requiring the full history of prior imaging reports. The model was tasked with classifying reports into five distinct categories: Baseline (BL), Complete Response (CR), Partial Response (PR), Stable Disease (SD), and Progressive Disease (PD).

The model was tasked with classifying reports into five distinct categories: Baseline (BL), Complete Response (CR), Partial Response (PR), Stable Disease (SD), and Progressive Disease (PD). While RECIST 1.1 defines BL as the initial reference timepoint rather than a response category, we included it as a classification target to ensure the model can robustly differentiate between treatment-naïve reference scans and subsequent follow-up assessments. This architectural choice is critical for the automated processing of longitudinal clinical data, as it prevents the model from erroneously assigning response status to initial examinations. To enable unbiased evaluation, explicit RECIST outcome labels (Baseline, Complete Response, Partial Response, Stable Disease, Progressive Disease) were programmatically removed from the text prior to processing. A reference set of original classifications, as documented in the reports, was retained for performance benchmarking.

We evaluated the offline performance of the LLaMA 3.3–70B large language model, deployed securely within institutional infrastructure to ensure full compliance with data privacy requirements. No model fine-tuning was applied. Instead, we systematically examined the effectiveness of three prompting strategies for guiding the model’s tumor response classification:

- Zero-shot prompting.

**Table:** 

Role	Content (exact text)
System	“You are a board-certified oncologic radiologist. Answer only with one of the following tokens (no explanations): CR, PR, SD, PD, BL.”
User	[Verbatim content of the structured RECIST report]
Rationale	The model receives no examples and is forced to infer the label solely from the clinical context.

Inference settings. temperature = 0.0, top_p = 0.9. The first token matching the regex \b(CR|PR|SD|PD|BL)\b was parsed as prediction; all other text was discarded.

- Few-shot prompting.

**Table:** 

Role	Content
System	“You are an oncologic radiologist. Return valid JSON exactly like {“recist”:“CR”} using one of “CR”,“PR”,“SD”,“PD”,“BL”.”
User / Assistant (×2–5 per class)	Alternating pairs of < RECIST report excerpt ≤ 450 chars > and t he corresponding assistant reply {“recist”:“< gold label>”}.
User (query)	[Full RECIST report to be classified]
Rationale	Few-shot in-context learning supplies two-five prototypical reports for every label, exposing the required JSON format

Same decoding as above. Examples were sampled once with a fixed random seed and held constant for all test cases.

- Chain-of-thought prompting.

**Table:** 

Role	Content
System	“You are an oncologic radiologist. Think step-by-step in plain English. After you finish reasoning, output the final RECIST category on a new line prefixed with ANSWER: using one of CR, PR, SD, PD, BL.”
User	[Full RECIST report]
For every report, *N* = 5 independent reasoning traces were sampled (temperature = 0.7, top_p = 0.95)	

The majority label after stripping the final line (ANSWER: < token> ) was taken as the prediction.

Chain-of-thought elicits decomposed reasoning; self-consistency aggregates multiple stochastic traces, mitigating “lucky” or “hallucinated” chains.

We used an explicit chain-of-thought prompting strategy in which the model generated step-by-step reasoning in natural language. For evaluation, only the final RECIST label was extracted; intermediate reasoning traces were discarded and not stored. Performance metrics included accuracy, precison, recall as well as micro and macro F1 scores. Analyses were conducted using Python (NumPy 1.26.4, pandas 2.2.0, scikit-learn 1.4.0, statsmodels 0.14.1, matplotlib 3.8.2, seaborn 0.13.2)^17–22^. Per-class F1 score is computed independently for each class based on its precision and recall. This highlights how well the model performs on each individual category. Micro F1 score was calculated by aggregating the total true positives, false positives, and false negatives across all classes before computing the F1 score. This metric is weighted by class frequency and is more influenced by the model’s performance on the most common classes. Macro F1 score is the arithmetic mean of all per-class F1 scores. Each class contributes equally, regardless of its frequency, making this metric sensitive to poor performance on minority classes. To quantify statistical uncertainty, 95% confidence intervals (CIs) for accuracy and F1-scores were estimated using non-parametric bootstrapping with 3000 resamples at the report level. For each bootstrap iteration, metrics were recalculated based on the resampled dataset, and percentile-based confidence intervals (2.5th–97.5th percentiles) were derived.

### Code availability

The custom computer code used to generate the results and perform the statistical analyses in this study is provided as a Supplementary Information file accompanying this manuscript. The code includes the implementation of the zero-shot, few-shot, and chain-of-thought prompting strategies using the LLaMA-3.3 (70B) model as described in the Methods section.

## Results

### Overall model performance

**Fig. 1:**
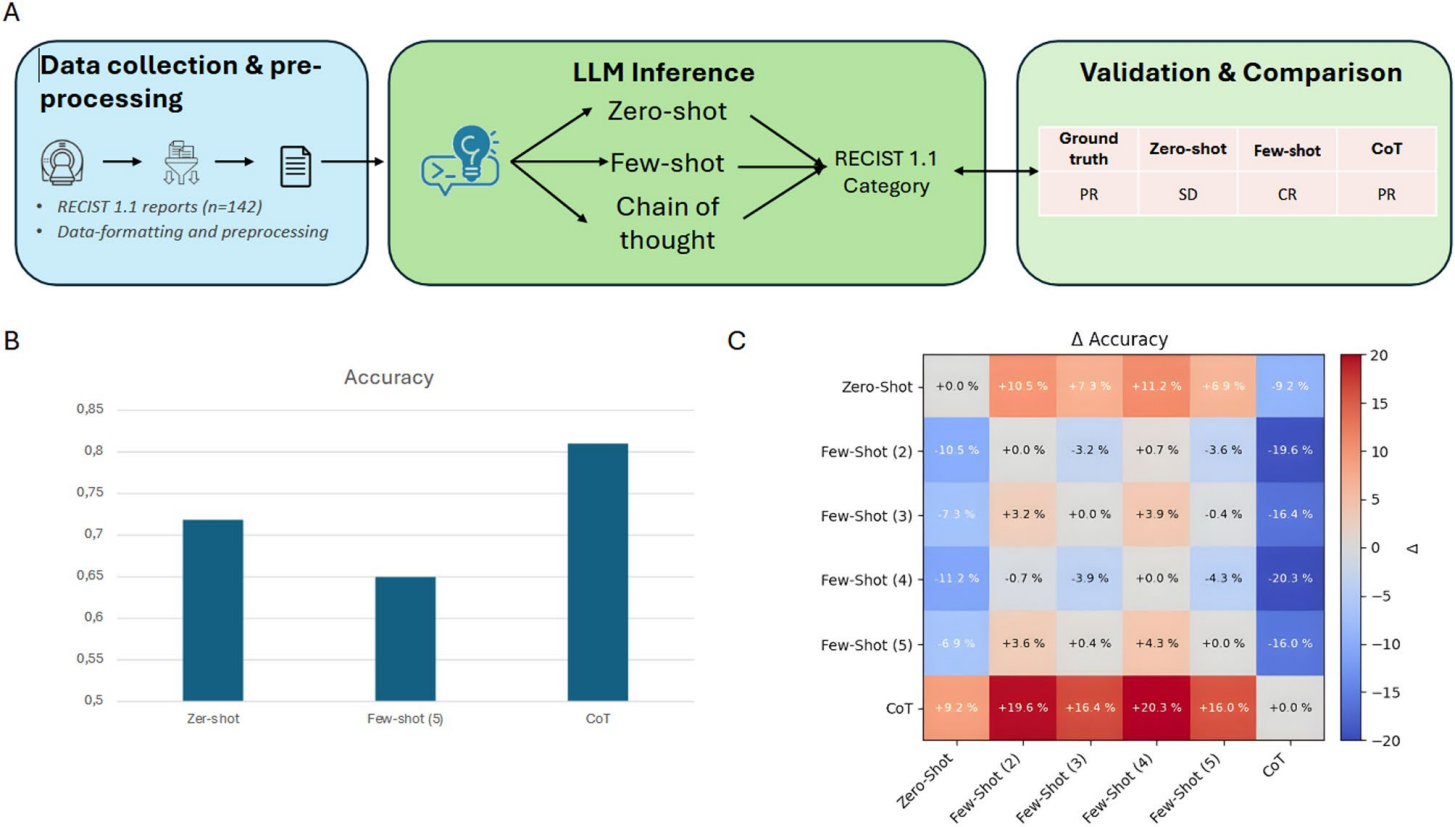
(A) Schematic overview of the classification workflow for RECISTI reports. After report acquisition and data pre-processing, Llama 3.3(70b) was prompted in a zero-, few-shot and chain of thought manner to predict RECIST classification. (B) Bar chart illustrating the accuracy scores for accurate RECIST classification (C) Heatmap depicting pairwise performance differences for each prompting strategy used. Positive (red) cells indicate a higher performance for the first named compared to the stronnamed strategy, while negative (blue) cells indicate lower performance.

We benchmarked three prompting strategies—zero-shot, few-shot with 2 / 3 / 4 / 5 examples per class, and Chain-of-Thought + Self-Consistency (CoT + SC)—on 142 structured RECIST reports (117 for the 5-shot setting owing to hold-out of support examples). The experimental workflow can be seen in Fig. 1A.

CoT + SC achieved the best micro-F1 (0.81) and accuracy (0.810), exceeding the next-best approach (zero-shot, accuracy = 0.72) by an absolute + 9% points. (Fig. 1B). Pairwise performance differences can be seen in Fig. 1C.

### Class-wise performance

Beyond overall accuracy, we assessed class-specific metrics—precision, recall and F1-score—for all five RECIST outcome categories: CR (Complete Response), PR (Partial Response), SD (Stable Disease), PD (Progressive Disease), and BL (Baseline). This analysis allows a more granular understanding of how well each prompting strategy handles different response types, especially those that are underrepresented or harder to classify.

In the Zero-Shot condition, performance varied markedly across classes. The model achieved near-perfect precision and high recall for the “BL” class (precision = 1.000, recall = 0.844), suggesting that baseline reports are easily identified even without prior examples. The “PR” class, which comprised the largest proportion of samples, also showed solid performance (F1 = 0.748). In contrast, the “CR” class was problematic (F1 = 0.500), reflecting difficulty in distinguishing complete response from similar categories without contextual anchoring.

The Few-Shot (k = 5) model showed improved balance for rare classes like “CR” and “PD”. While the “BL” category retained perfect precision, the model showed slightly reduced recall (0.870), likely due to overfitting to few-shot examples. The “PD” class achieved an F1 of 0.889—up from 0.714 in zero-shot—highlighting the benefits of in-context supervision. However, the “PR” class suffered a small drop in both recall and precision, suggesting that few-shot performance is sensitive to example selection, particularly in the presence of label imbalance.

The best results were achieved with (CoT + SC). All classes improved across at least one metric. Notably, the F1-score for “CR” rose to 0.588, while “SD” increased sharply from 0.519 (zero-shot) to 0.786, driven primarily by a 54-percentage-point gain in recall. The “PR” class reached an F1 of 0.909, the highest among all settings, indicating that CoT reasoning helps disambiguate partial vs. complete response. Interestingly, the model appeared slightly more conservative in assigning “BL” labels (recall = 0.531), potentially due to more nuanced internal reasoning chains that defer to treatment-related labels.

Across all prompting strategies, precision remained generally higher than recall. The macro-averaged F1 score was highest under CoT + SC (0.762), further supporting its robustness across label types.

Together, these results emphasize that class-level behavior varies meaningfully with prompting strategy. Incorporating reasoning and ensemble mechanisms (as in CoT + SC) not only improves aggregate performance but also mitigates errors in edge cases like “CR” and “SD”, which are often underrepresented and semantically adjacent to other categories. Results can be seen in Tables 1, 2 and 3.

**Table 1:** Zero-shot, few-shot (5) and Chain of thought prediction performance with Data are precision, recall, F1-score and accuracy.

Class	Precision	Recall	F1-Score	*n*
BL	1	0,844	0,915	32
CR	0,357	0,833	0,5	6
PD	0,588	0,909	0,714	11
PR	0,708	0,793	0,748	58
SD	0,737	0,4	0,519	35
Accuracy			0,718 [0,641 – 0,789]	142
Macro avg	0,678	0,756	0,679 [0,581 – 0,760]	142
Micro avg	0,757	0,718	0,716 [0,641 – 0,789]	142

The F1 score was calculated as the harmonic mean of precision (also known as positive predictive value) and recall (also known as sensitivity). The micro scores were computed by aggregating the true-positive, false-negative, and false-positive findings across all classes. The macro scores was computed by calculating the scores for each class individually and then averaging them, giving equal weight to each class regardless of its size.

**Table 2:** Zero-shot, few-shot (5) and Chain of thought prediction performance with Data are precision, recall, F1-score and accuracy.

Class	Precision	Recall	F1-Score	*n*
BL	1	0,87	0,93	23
CR	0,053	1	0,1	1
PD	1	0,8	0,889	10
PR	0,643	0,551	0,593	49
SD	0,714	0,588	0,645	34
Accuracy			0,65 [0,564 – 0,735]	117
Macro avg	0,682	0,762	0,632 [0,555 – 0,700]	117
Micro avg	0,759	0,65	0,696 [0,564 – 0,735]	117

The F1 score was calculated as the harmonic mean of precision (also known as positive predictive value) and recall (also known as sensitivity). The micro scores were computed by aggregating the true-positive, false-negative, and false-positive findings across all classes. The macro scores was computed by calculating the scores for each class individually and then averaging them, giving equal weight to each class regardless of its size.

**Table 3:** Zero-shot, few-shot (5) and Chain of thought prediction performance with Data are precision, recall, F1-score and accuracy.

Class	Precision	Recall	F1-Score	*n*
BL	1	0,531	0,694	32
CR	0,455	0,833	0,588	6
PD	0,769	0,909	0,833	11
PR	0,962	0,862	0,909	58
SD	0,673	0,943	0,786	35
Accuracy			0,81 [0,746 – 0,873]	142
Macro avg	0,772	0,816	0,762 [0,664 – 0,844]	142
Micro avg	0,863	0,81	0,811 [0,746 – 0,873]	142

The F1 score was calculated as the harmonic mean of precision (also known as positive predictive value) and recall (also known as sensitivity). The micro scores were computed by aggregating the true-positive, false-negative, and false-positive findings across all classes. The macro scores was computed by calculating the scores for each class individually and then averaging them, giving equal weight to each class regardless of its size.

### Error topology

**Fig. 2:**
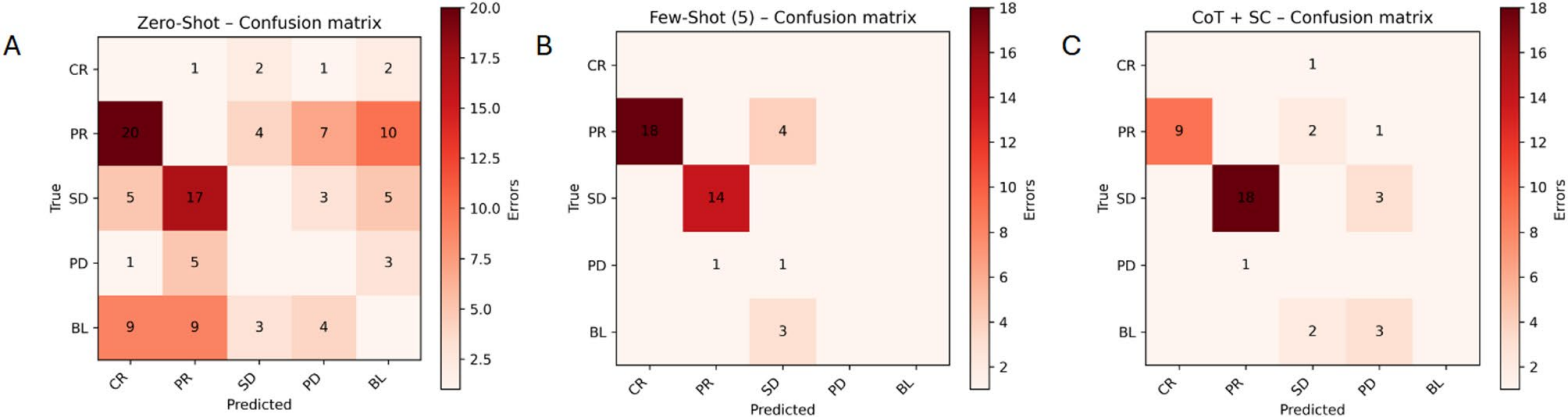
Confusion matrices comparing the multi-class classification performance of three prompting approaches—zero-shot, few-shot (5) and CoT. Each matrix plots the true labels on the y-axis against the predicted labels on the x-axis, with color intensity corresponding to the number of instances in each cell (darker shades indicate higher counts).

Figure 2 displays mis-classification heat-maps (correct predictions were masked).

To better understand the types and structure of classification errors across prompting strategies, we analyzed confusion matrices with a focus on misclassification patterns—that is, which classes are most often confused with one another, and how these patterns change with prompting strategy.

In the Zero-Shot condition, misclassifications were predominantly concentrated among the semantically adjacent categories of “PR” (partial response), “SD” (stable disease), and “CR” (complete response). The model frequently predicted “PR” when the true label was “SD”, and vice versa. Notably, some “CR” cases were misclassified as “BL” or “PR”, suggesting that the model struggled to differentiate complete response from less clear or incomplete responses. The confusion matrix also revealed false positives in “CR” predictions, which aligns with the low precision score (0.357) for this class. These patterns suggest that, in the absence of contextual anchoring, the model relies heavily on surface-level textual features, which may be ambiguous or insufficient for nuanced categorization.

The Few-Shot (5 examples per class) strategy slightly shifted the error topology but did not fundamentally resolve these ambiguities. While the recall for “CR” improved (1.000), the precision deteriorated even further (0.053), indicating that nearly every “CR” prediction was a false positive. This is visible in the confusion matrix, where off-diagonal cells related to “CR” predictions are prominently populated. Moreover, “PR” and “SD” remained the most confused pair, although some confusion shifted toward the “BL” (baseline) class, likely due to limited variation in the few-shot examples and the rigid format imposed by the few-shot prompt. Compared to zero-shot, few-shot learning reduced some of the spurious “CR”→”BL” errors, but introduced new inconsistencies, especially for “PR” cases.

In contrast, the Chain-of-Thought + Self-Consistency (CoT + SC) approach demonstrated a more structured and clinically plausible error topology. The confusion matrix revealed a substantial reduction in false positives across all classes, particularly for “CR” and “SD”. For instance, “CR” predictions were far more targeted, with fewer misclassifications into “PR” or “BL”. The model also showed strong alignment on “PR”, correctly identifying the majority of such cases with minimal overlap into “SD” or “PD”. Errors were sparser and more symmetrically distributed, with fewer high-density off-diagonal cells, indicating that the model was less prone to systematic biases toward specific classes. Notably, the “SD” class, which had been frequently confused in the previous strategies, achieved both high recall (0.943) and an error profile suggesting greater certainty in borderline decisions.

These confusion matrix patterns are not only quantitatively reflected in the F1-scores but also qualitatively illustrative of the model’s behavior. While Zero-Shot and Few-Shot predictions often show diagonal asymmetries and class-specific clustering of errors, CoT + SC exhibits a more diagonal-dominant, low-noise matrix, aligning well with clinical reasoning. Figures X–Y (see attached heatmaps) visualize these topologies, highlighting both the frequency and nature of classification errors that each prompting approach produces.

### Statistical comparison

Pair-wise McNemar tests confirmed that CoT + SC significantly outperformed both baselines after Bonferroni correction (Table 4). No significant difference was observed between zero-shot and few-shot (5).

**Table 4:** Statistical comparison of classification performance between prompting strategies using McNemar’s test.

Comparison	*p*-value	Corrected *p*-value
Zero-Shot vs. Few-Shot (5)	0.4725	1.000
Zero-Shot vs. CoT + SC	0.0000	0.0000
Few-Shot (5) vs. CoT + SC	0.0000	0.0000

### Shot-count ablation

To evaluate the impact of the number of in-context examples (“shots”) on model performance, we conducted a shot count ablation by varying the number of examples per class in the few-shot prompting setup from k = 0 (i.e., zero-shot) to k = 5. For each configuration, we measured micro-averaged F1-scores across all classes to assess overall performance sensitivity to the number of shots.

The results reveal a non-linear relationship between the number of shots and model effectiveness. The zero-shot baseline achieved a micro-F1 of 0.716, which served as a surprisingly strong reference point. Adding just two examples per class (k = 2) led to a decrease in performance (micro-F1 = 0.652), indicating that limited contextual examples may introduce overfitting or noise, especially in a setting with imbalanced class distributions and variable linguistic structure in clinical texts.

As the shot count increased, performance gradually recovered. With k = 3, the micro-F1 rose to 0.693, and at k = 4, it stabilized at 0.654. The highest few-shot result was observed at k = 5 (micro-F1 = 0.696), but still did not surpass the zero-shot baseline, suggesting diminishing returns beyond a certain point. Notably, none of the few-shot configurations matched or outperformed the Chain-of-Thought + Self-Consistency approach, which achieved a micro-F1 of 0.811, highlighting the advantage of incorporating structured reasoning over increasing the number of raw examples.

These findings suggest that more examples do not necessarily lead to better performance in this domain. Instead, the quality, diversity, and representativeness of few-shot examples may be more critical than their quantity. Moreover, the fact that zero-shot prompting outperformed all k-shot configurations in this study reinforces the importance of prompt design and model reasoning capability over brute-force in-context loading.

**Fig. 3:**
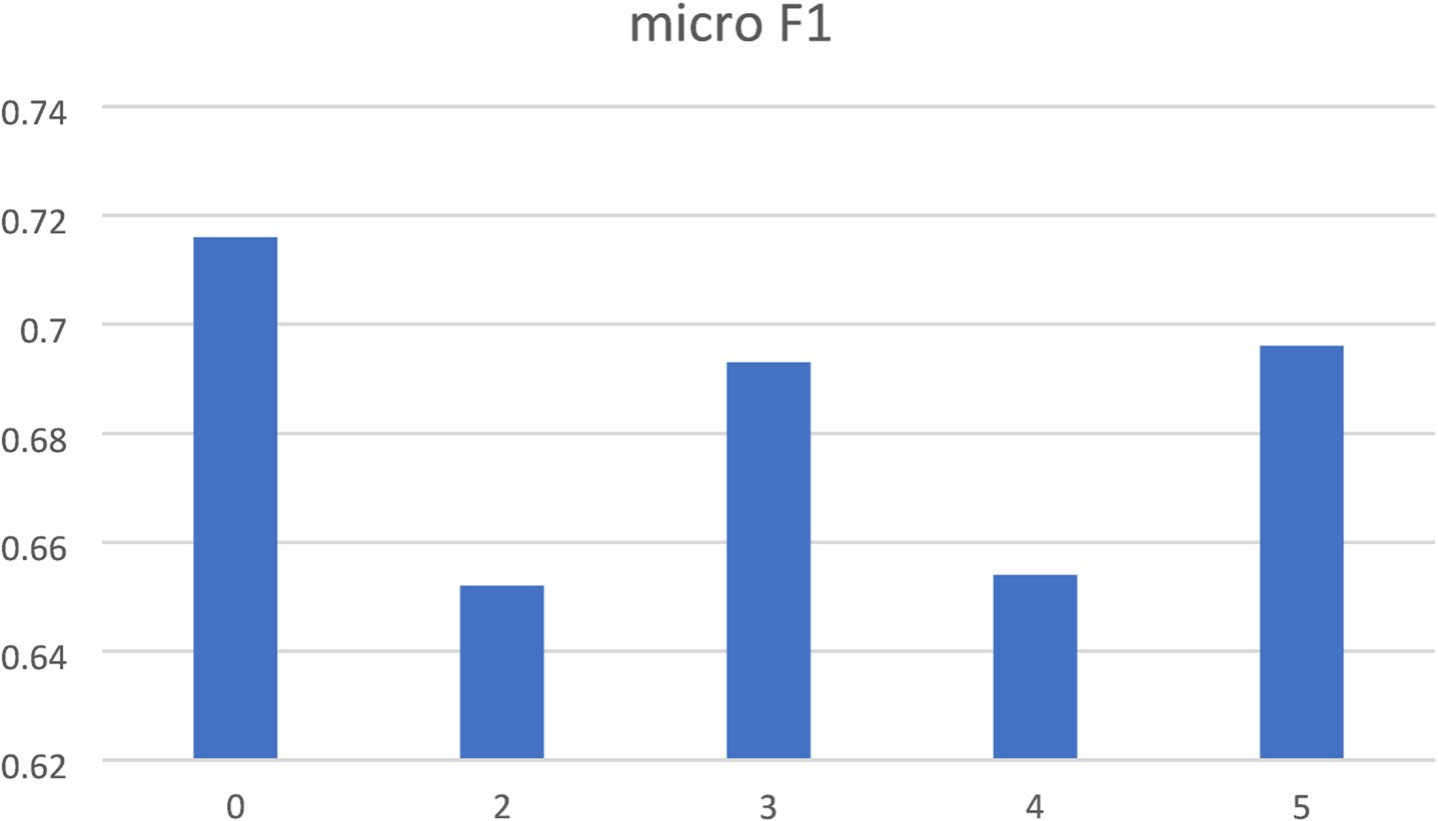
Shot count ablation analysis for few-shot prompting. The bar chart illustrates the impact of increasing the number of in-context examples per class (k) on micro-averaged F1-score in the RECIST classification task.

Ablation results are summarized in Fig. 3, showing micro-F1 scores across shot counts and reinforcing the conclusion that effective prompting strategy selection outweighs simple scaling of in-context examples in structured medical classification tasks.

## Discussion

This study demonstrates the feasibility and effectiveness of using prompt-guided large language models (LLMs) for the automated classification of RECIST based tumor response classification, based on routine CT imaging reports. By applying multiple prompting strategies to an offline deployment of LLaMA 3.3–70B, we show that general-purpose foundation models can accurately map structured radiology findings to standardized RECIST outcome categories.

It is important to acknowledge the distinct types of variability in oncologic response assessment. As noted in recent literature, a primary source of interobserver variability in RECIST 1.1 is the radiologist’s selection of target lesions^23,24^. Since our model operates downstream of the image interpretation and lesion measurement process, it does not mitigate variability arising from target lesion selection. Instead, it addresses ‘interpretive variability’ and documentation errors. In clinical practice, discordance can occur where the measurements indicate one category (e.g., ‘Progressive Disease’ based on Nadir), but the free-text conclusion erroneously states ‘Stable Disease’ due to calculation errors or cognitive bias. By automating the mapping of findings to categories, the LLM acts as a consistency check for the reporting radiologist.

Unlike many prior studies that rely on simulated or synthetic data^15,25,26^ our work is grounded in real-world clinical documentation, reflecting the variability and nuance of routine oncology practice. Reports originated from diverse imaging contexts, were authored by practicing radiologists, and adhered to institutionally standardized, but stylistically varied, RECIST formats. This diversity posed a meaningful challenge for natural language understanding, yet the LLaMA 3 model was able to generalize across these differences when guided by well-crafted prompts. Notably, high F1 scores across categories and strong agreement with expert labels indicate that prompting alone can yield clinically relevant classification performance.

Our comparison of prompting strategies offers insights into how different interaction modes affect model behavior. While zero-shot prompting provided a strong baseline, chain-of-thought reasoning further enabled contextual alignment by injecting targeted examples without hardcoding specific rules or templates. These findings support the idea that prompt engineering can serve as a flexible alternative to traditional model fine-tuning, particularly in secure environments where data cannot leave institutional boundaries. However, prompt engineering may be sensitive to subtle changes in wording or context and lacks the task-specific optimization that fine-tuned models can offer.

Previous AI applications in oncology have predominantly focused on structured data^27,28^. More recent advances have explored LLMs in information extraction from pathology and imaging narratives, often within narrower clinical domains such as breast or lung cancer^29–33^. By extending this paradigm to the task of RECIST classification in general solid tumors, using real-world radiology reports, our work contributes a broader validation of LLM utility in oncology text analysis.

Nevertheless, certain limitations merit attention. First, this study followed a retrospective design and evaluated model performance exclusively on previously finalized radiology reports, which limits conclusions regarding real-time clinical deployment or prospective decision support. In addition, while we evaluated the model on a sizable clinical dataset, the scope was limited to reports from a single institution. The use of single-center data may restrict generalizability due to stylistic homogeneity in report language and local reporting conventions. Moreover, all reports adhered to institutionally standardized RECIST-structured reporting, which likely facilitated model interpretation and may limit transferability to settings where reports are less structured or entirely free-text. Multi-center datasets with broader linguistic and stylistic variability are therefore needed to validate robustness and generalizability. Second, despite strong overall performance, some misclassifications occurred in borderline cases, particularly when narrative expressions were vague or non-standardized. The observed confusion between Complete Response (CR) and Baseline (BL) or Partial Response (PR) underscores the complexity of temporal anchoring in oncology. While “no residual disease” is a hallmark of CR, similar phrasing may appear in baseline reports of patients post-resection or in adjuvant settings. The model’s errors in these “CR” cases often occurred when it failed to distinguish whether the absence of disease was the reference starting point (Baseline) or the result of the current systemic therapy (CR). This highlights that for automated systems, understanding the clinical ‘intent’ and the specific treatment phase is as vital as parsing the measurements themselves. Incorporating feedback loops or uncertainty estimation mechanisms may improve interpretability and trustworthiness in clinical settings. Incorporating feedback loops or uncertainty estimation mechanisms may improve interpretability and trustworthiness in clinical settings. Furthermore, our classification represents a point-in-time interpretation of individual radiology reports. In a formal clinical trial setting, RECIST 1.1 requires confirmation of response (for CR and PR) through follow-up imaging. Additionally, the nuances of non-measurable disease, where response assessment relies on the presence or absence of unequivocal progression, pose a higher challenge for automated systems. Future iterations of the model could be designed to incorporate longitudinal data (i.e., comparing current and prior reports) to better align with the formal requirement for response confirmation and specialized progression criteria. Finally, while our model operated entirely offline, which represents a strength with respect to data privacy, it also limited the ability to dynamically update retrieval sources or integrate external medical knowledge bases in real time.

Future work should include prospective validation and evaluation in workflow-integrated settings, such as RIS or PACS environments, to assess real-time performance and clinical usability. In addition, role-based prompting strategies may further enhance model alignment with clinical reporting standards by explicitly instructing the model to adopt the perspective of an oncologic radiologist applying RECIST 1.1 criteria. Such approaches could strengthen interpretive consistency while preserving data privacy and avoiding the need for task-specific fine-tuning.

From an operational perspective, the approach can be deployed entirely within institutional infrastructure and does not rely on real-time inference. Model execution can occur in the background, making processing delays at the level of individual reports acceptable for non-time-critical clinical workflows such as retrospective analysis or quality assurance.

In potential clinical applications, outputs generated by the model should be interpreted as supportive information and remain subject to review by qualified clinicians. In cases of uncertainty, ambiguous findings, or disagreement between model outputs and clinical judgment, human review and final decision-making are essential. Such a human-in-the-loop setup ensures that responsibility for interpretation remains with the clinician and helps mitigate risks associated with incorrect or overconfident model predictions. Accordingly, the approach is best viewed as an assistive tool rather than an autonomous decision-making system.

This study highlights the utility of prompt-driven LLMs for structured tumor response classification in oncology, using real, unmodified CT reports. The ability to accurately extract RECIST outcomes from narrative text—without retraining or external data access—offers a practical and privacy-preserving path toward automating parts of the oncology reporting workflow. Future research should explore multi-institutional validation, real-time clinical integration, and clinician-in-the-loop strategies to enhance robustness and adoption in daily practice. To support clinical uptake, future systems could embed prompt-guided LLMs into PACS/RIS environments for real-time feedback during report drafting. These findings further illustrate how LLMs, when deployed thoughtfully, can contribute meaningfully to scalable, reproducible, and interpretable decision support in cancer care.

## Conclusion

This study demonstrates that prompt-driven large language models can effectively classify tumor response according to RECIST criteria using real-world structured CT radiology reports. By leveraging structured prompting strategies without requiring model fine-tuning or external data access, our approach enables accurate, scalable, and privacy-preserving automation of oncology reporting workflows. The use of LLMs in this context offers a promising step toward reducing manual workload and ensuring that text-based conclusions strictly adhere to the mathematical criteria of RECIST, thereby supporting decision-making in cancer care. Future work should focus on validating these findings across multiple institutions, integrating such models into real-time reporting systems, and developing clinician-in-the-loop mechanisms to ensure safe and effective clinical deployment.

## Supplementary Information

Below is the link to the electronic supplementary material.

Supplementary Material 1

## Data Availability

The datasets generated and/or analysed during the current study are not publicly available due to patient data protection and institutional regulations but are available from the corresponding author on reasonable request.

## References

[1] Eisenhauer, E. A. et al. New response evaluation criteria in solid tumours: revised RECIST guideline (version 1.1). *Eur. J. Cancer*. **45** (2), 228–247. 10.1016/j.ejca.2008.10.026 (2009). 19097774 10.1016/j.ejca.2008.10.026

[2] Xu, Z., Jiang, G. & Dai, J. Tumor therapeutics in the era of RECIST: past, current insights, and future prospects. *Oncol. Rev.* **18**, 1435922. 10.3389/or.2024.1435922 (2024). 39493769 10.3389/or.2024.1435922 PMC11527623

[3] Moor, M. et al. Foundation models for generalist medical artificial intelligence. *Nature* **616** (7956), 259–265 (2023). 37045921 10.1038/s41586-023-05881-4

[4] Hager, P. et al. Evaluation and mitigation of the limitations of large language models in clinical decision-making. *Nat. Med.* **30**, 2613–2622. 10.1038/s41591-024-03097-1 (2024). 38965432 10.1038/s41591-024-03097-1 PMC11405275

[5] Busch, F. et al. Large language models for structured reporting in radiology: past, present, and future. *Eur. Radiol.* (2024). 10.1007/s00330-024-11107-6 PMC12021971 39438330

[6] Menezes, M. C. S. et al. The potential of Generative Pre-trained Transformer 4 (GPT-4) to analyse medical notes in three different languages: a retrospective model-evaluation study. *Lancet Digit. Health*. **7** (1), e35–e43 (2025). 39722251 10.1016/S2589-7500(24)00246-2 PMC12182955

[7] Eguia, H., Sánchez-Bocanegra, C., Vinciarelli, F., Alvarez-Lopez, F. & Saigí-Rubió, F. Clinical decision support and natural language processing in medicine: systematic literature review. *J. Med. Internet. Res.* **26**, e55315. 10.2196/55315 (2024). 39348889 10.2196/55315 PMC11474138

[8] Wu, C. et al. Towards evaluating and building versatile large language models for medicine. *npj Digit. Med.* **8**, 58. 10.1038/s41746-024-01390-4 (2025). 39865143 10.1038/s41746-024-01390-4 PMC11770143

[9] Reichenpfader, D., Muller, H. & Denecke, K. A scoping review of large language model-based approaches for information extraction from radiology reports. *NPJ Digit. Med.* **7** (1), 222 (2024). 39182008 10.1038/s41746-024-01219-0 PMC11344824

[10] Bhattarai, K. et al. Leveraging GPT-4 for identifying cancer phenotypes in electronic health records: a performance comparison between GPT-4, GPT-3.5-turbo, Flan-T5, Llama-3-8B, and spaCy’s rule-based and machine learning-based methods. *JAMIA Open.* **7** (3), ooae060 (2024). 38962662 10.1093/jamiaopen/ooae060 PMC11221943

[11] Bhagat, N., Mackey, O. & Wilcox, A. Large language models for efficient medical information extraction. In *AMIA Joint Summits on Translational Science Proceedings, 2024* 509–514 (2024). PMC11141860 38827084

[12] Wang, Y. & Rajkomar, A. Large language models-powered clinical decision support. *Intelli.-Based Med.* **11**, 100123. 10.1016/j.imed.2025.01.001 (2025).

[13] Gaber, F. et al. Evaluating large language model workflows in clinical decision support for triage and referral and diagnosis. *npj Digit. Med.* **8**, 263. 10.1038/s41746-025-01684-1 (2025). 40346344 10.1038/s41746-025-01684-1 PMC12064692

[14] Yang, X. et al. GatorTron: a large clinical language model to unlock patient information from unstructured electronic health records. *npj Digit. Med.* **5**, 62. 10.1038/s41746-022-00742-2 (2022).35551275

[15] Spitzl, D. et al. Leveraging large language models for accurate classification of liver lesions from MRI reports. *Comput. Struct. Biotechnol. J.* **23**,100182. 10.1016/j.csbj.2025.05.019 (2025). 10.1016/j.csbj.2025.05.019 PMC12158552 40502931

[16] Kanzawa, J. et al. Automated classification of brain MRI reports using fine-tuned large language models. *Neuroradiology* **66** (12), 2177–2183 (2024). 38995393 10.1007/s00234-024-03427-7 PMC11611921

[17] Harris, C. R. et al. Array programming with NumPy. *Nature* **585**, 357–362 (2020). 32939066 10.1038/s41586-020-2649-2 PMC7759461

[18] McKinney, W. Data structures for statistical computing in Python. In *Proceedings of the 9th Python in Science Conference* 56–61 (2010).

[19] Pedregosa, F. et al. Scikit-learn: machine learning in Python. *J. Mach. Learn. Res.* **12**, 2825–2830 (2011).

[20] Hunter, J. Matplotlib: a 2D graphics environment. *Comput. Sci. Eng.* **9**, 90–95 (2007).

[21] Seabold, S. & Perktold, J. Statsmodels: econometric and statistical modeling with Python. In *Proceedings of the 9th Python in Science Conference* (2010).

[22] Waskom, M. Seaborn: statistical data visualization. *J. Open. Source Softw.* **2021**, 66 (2021).

[23] Kuhl, C. K. et al. Validity of RECIST version 1.1 for response assessment in metastatic cancer: a prospective, multireader study. *Radiology* **290** (2), 349–356. 10.1148/radiol.2018180648 (2019). 30398433 10.1148/radiol.2018180648

[24] Tareco Bucho, T. M. et al. How does target lesion selection affect RECIST? A computer simulation study. *Invest. Radiol.* **59** (6), 465–471 (2024). 10.1097/RLI.0000000000001045 37921780

[25] Gu, K. et al. Using GPT-4 for LI-RADS feature extraction and categorization with multilingual free-text reports. *Liver Int.* **44** (7), 1578–1587. 10.1111/liv.15891 (2024). 38651924 10.1111/liv.15891

[26] Ruiz Sarrias, O. et al. Leveraging large language models for precision monitoring of chemotherapy-induced toxicities: a pilot study with expert comparisons and future directions. *Cancers (Basel)*. **16** (16), 2830. 10.3390/cancers16162830 (2024). 39199603 10.3390/cancers16162830 PMC11352281

[27] Nerella, S. et al. Transformers and large language models in healthcare: a review. *Artif. Intell. Med.* **154**, 102900. 10.1016/j.artmed.2024.102900 (2024). 38878555 10.1016/j.artmed.2024.102900 PMC11638972

[28] Du, X. et al. Enhancing early detection of cognitive decline in the elderly: a comparative study utilizing large language models in clinical notes. *EBioMedicine* **109**, 105401. 10.1016/j.ebiom.2024.105401 (2024). 39396423 10.1016/j.ebiom.2024.105401 PMC11663780

[29] Huang, J. et al. A critical assessment of using ChatGPT for extracting structured data from clinical notes. *NPJ Digit. Med.* **7** (1), 106. 10.1038/s41746-024-01079-8 (2024). 38693429 10.1038/s41746-024-01079-8 PMC11063058

[30] Fervers, P. et al. ChatGPT yields low accuracy in determining LI-RADS scores based on free-text and structured radiology reports in German language. *Front. Radiol.* **4**, 1390774 (2024). 39036542 10.3389/fradi.2024.1390774 PMC11257913

[31] Tozuka, R. et al. Application of NotebookLM, a large language model with retrieval-augmented generation, for lung cancer staging. *Japanese J. Radiol.* **43** (4), 706–712. 10.1007/s11604-024-01705-1 (2025). 10.1007/s11604-024-01705-1 39585559

[32] Mergen, M. et al. Leveraging large language models for accurate AO fracture classification from CT text reports. *J. Digit. Imaging Inf. med.*10.1007/s10278-025-01603-6 (2025). 10.1007/s10278-025-01603-6 PMC13103119 40624390

[33] Mergen, M. et al. LLM-powered TNM staging of neuroendocrine tumors from PET/CT reports. *BMC Med. Imaging*. **26**, 50. 10.1186/s12880-025-02092-3 (2026). 10.1186/s12880-025-02092-3 PMC12838453 41437331

